# HIV-Tat immunization induces cross-clade neutralizing antibodies and CD4^+^ T cell increases in antiretroviral-treated South African volunteers: a randomized phase II clinical trial

**DOI:** 10.1186/s12977-016-0261-1

**Published:** 2016-06-09

**Authors:** Barbara Ensoli, Maphoshane Nchabeleng, Fabrizio Ensoli, Antonella Tripiciano, Stefania Bellino, Orietta Picconi, Cecilia Sgadari, Olimpia Longo, Lara Tavoschi, Daniel Joffe, Aurelio Cafaro, Vittorio Francavilla, Sonia Moretti, Maria Rosaria Pavone Cossut, Barbara Collacchi, Angela Arancio, Giovanni Paniccia, Anna Casabianca, Mauro Magnani, Stefano Buttò, Elise Levendal, John Velaphi Ndimande, Bennett Asia, Yogan Pillay, Enrico Garaci, Paolo Monini, SMU-MeCRU study group

**Affiliations:** National AIDS Center, Istituto Superiore di Sanità, Rome, Italy; MeCRU, Sefako Makgatho Health Sciences University (SMU), Ga-Rankuwa, South Africa; Laboratory of Clinical Pathology and Microbiology, San Gallicano Institute, Istituti Fisioterapici Ospitalieri, Rome, Italy; Head Office, National AIDS Center, Istituto Superiore di Sanità, Cape Town, South Africa; Department of Biomolecular Science, University of Urbino, Urbino, Italy; South African Medical Research Council, Cape Town, South Africa; Department of Health, Marshall Town, Gauteng Province South Africa; National Department of Health, Pretoria, South Africa; Istituto Superiore di Sanità, Rome, Italy; National Center for Epidemiology, Surveillance and Health Promotion, Istituto Superiore di Sanità, Rome, Italy; Italian Medicines Agency, Rome, Italy; European Center for Disease Prevention and Control, Stockholm, Sweden; Health Systems Trust, Cape Town, South Africa; University of Tor Vergata, Rome, Italy

**Keywords:** Tat, HIV, AIDS, Clinical trials, Vaccine, Cross-clade antibodies, Neutralization, CD4^+^ T cells, cART, Therapy intensification

## Abstract

**Background:**

Although combined antiretroviral therapy (cART) has saved millions of lives, it is incapable of full immune reconstitution and virus eradication. The transactivator of transcription (Tat) protein is a key human immunodeficiency virus (HIV) virulence factor required for virus replication and transmission. Tat is expressed and released extracellularly by infected cells also under cART and in this form induces immune dysregulation, and promotes virus reactivation, entry and spreading. Of note, anti-Tat antibodies are rare in natural infection and, when present, correlate with asymptomatic state and reduced disease progression. This suggested that induction of anti-Tat antibodies represents a pathogenesis-driven intervention to block progression and to intensify cART. Indeed Tat-based vaccination was safe, immunogenic and capable of immune restoration in an open-label, randomized phase II clinical trial conducted in 168 cART-treated volunteers in Italy. To assess whether B-clade Tat immunization would be effective also in patients with different genetic background and infecting virus, a phase II trial was conducted in South Africa.

**Methods:**

The ISS T-003 was a 48-week randomised, double-blinded, placebo-controlled trial to evaluate immunogenicity (primary endpoint) and safety (secondary endpoint) of B-clade Tat (30 μg) given intradermally, three times at 4-week intervals, in 200 HIV-infected adults on effective cART (randomised 1:1) with CD4^+^ T-cell counts ≥200 cells/µL. Study outcomes also included cross-clade anti-Tat antibodies, neutralization, CD4^+^ T-cell counts and therapy compliance.

**Results:**

Immunization was safe and well-tolerated and induced durable, high titers anti-Tat B-clade antibodies in 97 % vaccinees. Anti-Tat antibodies were cross-clade (all vaccinees tested) and neutralized Tat-mediated entry of oligomeric B-clade and C-clade envelope in dendritic cells (24 participants tested). Anti-Tat antibody titers correlated positively with neutralization. Tat vaccination increased CD4^+^ T-cell numbers (all participants tested), particularly when baseline levels were still low after years of therapy, and this had a positive correlation with HIV neutralization. Finally, in cART non-compliant patients (24 participants), vaccination contained viral load rebound and maintained CD4^+^ T-cell numbers over study entry levels as compared to placebo.

**Conclusions:**

The data indicate that Tat vaccination can restore the immune system and induces cross-clade neutralizing anti-Tat antibodies in patients with different genetic backgrounds and infecting viruses, supporting the conduct of phase III studies in South Africa.

*Trial registration* ClinicalTrials.gov NCT01513135, 01/23/2012

**Electronic supplementary material:**

The online version of this article (doi:10.1186/s12977-016-0261-1) contains supplementary material, which is available to authorized users.

## Background

South Africa is severely affected by human immunodeficiency virus (HIV) infection [1]. The HIV counselling and testing campaign (HCT) launched by the National Department of Health has steadily increased the proportion of HIV-infected patients on combined antiretroviral therapy (cART). However, access to therapy and care of millions of people living with HIV is posing an enormous challenge to the public health system by means of a growing work overload and economic burden. This is going to be further complicated by the expected implementation of the new World Health Organization (WHO) guidelines that recommend starting therapy at the time of the first positive HIV testing (“test and treat”) [2]. However, despite vast access to cART, the rates of HIV morbidity/mortality are still high, with a 14 % annual increase of HIV drug resistance related to insufficient treatment compliance, which hampers an effective suppression of virus replication, a prerequisite to reduce virus transmission [3]. Further, late therapy initiation is still frequent in South Africa limiting the extent of CD4^+^ T cell recovery and immune restoration [4, 5]. Similarly, persistent immune activation, particularly when associated with poor immunological response to therapy, leads to disease progression even under HIV suppression [6–8]. These are causes of increasing co-morbidities, hospitalization, deaths and costs for the National Health Systems. In this context, an effective therapeutic vaccine, in conjunction with existing strategies, may represent a relevant, cost-effective intervention to intensify cART [9].

The transactivator of transcription (Tat) is a key HIV virulence factor playing pivotal roles in virus gene expression, replication, transmission and disease progression (reviewed in [10, 11]). Tat is produced very early upon infection [12–16] and continues to be expressed under cART [17, 18], is released extracellularly [19–21], accumulates in tissues [22, 23], and exerts effects on both the virus and the immune system [17, 24–52] that make it an optimal candidate for therapeutic immunization and cART intensification [53–58]. In particular, by promoting an excessive and improper immune stimulation, Tat prepares target cells for virus propagation, while disabling an effective immune control [17, 24–52]. This leads to the chronic loss of immune homeostasis observed in HIV-infected patients, which is only partially reverted by cART [59–63]. Further, extracellular Tat, which is present on virions [64], binds the envelope (Env) spikes forming a virus entry complex that favors infection of dendritic cell (DC) and T cells, key components of the virus reservoir [65]. Of note, by binding the Env C–C chemokine receptor 5 (CCR5) co-receptor binding sites, Tat shields Env from anti-HIV antibodies (Abs), thus inhibiting virus neutralization, which, however, is restored by anti-Tat Abs [65]. Notably, anti-Tat Abs are uncommon in natural infection and, when present, correlate with the asymptomatic state, higher CD4^+^ T-cell number, lower viral load, and reduced disease progression [66–70]. This suggested that the induction of effective anti-Tat Abs represents a pathogenesis-driven intervention to block progression and to intensify cART efficacy.

After completion of randomised, placebo-controlled, double-blinded phase I trials with the biologically active HIV-1 B-clade Tat protein in HIV-infected and uninfected individuals in Italy [54–56], an open-label randomised exploratory phase II trial with Tat was conducted in 168 HIV-infected anti-Tat Abs negative, virologically suppressed cART-treated (mean of 6 years) adult subjects in Italy (ISS T-002, ClinicalTrials.gov NCT00751595) [53, 57]. The endpoints were to evaluate immunogenicity and safety of B-clade Tat protein administered at 7.5 or 30 µg, given three or five times monthly, and to investigate immunological and virological disease biomarkers. The vaccine was safe and well tolerated and induced anti-Tat Abs in most patients (79 %), with the highest frequency and durability in the Tat 30 µg groups (89 %), particularly when given 3 times (92 %). Vaccination promoted a durable and significant restoration of T, B, and natural killer (NK) cell numbers, increased CD4^+^ and CD8^+^ central memory subsets, and upregulated the expression of human leukocyte antigen-D related (HLA-DR^+^) on CD8^+^ killer T cells, a phenotype found to be increased in elite controllers and to contribute to HIV containment [71, 72]. Moreover, a significant reduction of blood proviral DNA was seen after 3 years from the first immunisation, particularly under protease inhibitor (PI)-based regimens and with Tat 30 µg given three times (30 μg, 3×), reaching a predicted 70 % decay with a half-life of 88 weeks [57]. This decay was significantly associated with anti-Tat immunoglobulin (Ig) M and IgG Ab titers and neutralization of Tat-mediated entry of oligomeric Env in DC. Neutralization predicted HIV-1 DNA decay [57].

Based on these data, a 48-week randomised, double-blinded, placebo-controlled phase II study was conducted in cART-treated South African adult volunteers to verify the immunogenicity and safety of the B-clade Tat vaccine in a population with a different genetic background and mainly infected with a C clade virus. Anti-Tat Abs were further characterised to explore cross-clade recognition and their capability of cross-neutralising Tat-mediated oligomeric Env entry in DC. CD4^+^ T-cell counts were monitored for the entire trial, and the relationship between neutralization and CD4^+^ T-cell counts, as well as between anti-Tat and anti-Env Ab titers and neutralization, were also examined.

## Methods

### Production and purification of the recombinant biologically active HIV-1 Tat protein for human use

The biologically active recombinant clade B HIV-1 Tat, selected as vaccine candidate for human use, is the 86 amino acid-long protein derived from the HTLV-IIIB strain (BH-10 clone) (Additional file 1: Figure S1). The protein was produced under good manufacturing practice (GMP) conditions by Diatheva-Avitech APU Srl, Fano (PU), Italy. Tat vialing, packaging and batch release was performed by Injectalia Srl, Rome, Italy. Briefly, the Tat protein is obtained from a lysate of *Escherichia coli* cells engineered with the pET-tat plasmid, constructed for Tat expression. The pET system is based on the T7 promoter-driven system originally developed by Studier and colleagues [73–75], and provides vector-host combinations that enable tuning of basal expression levels to optimize target gene expression [75]. The GMP protein is then purified by diethylaminoethyl (DEAE) chromatography followed by heparin Sepharose chromatography. Following purification, the Tat protein is formulated in potassium phosphate saline buffer, pH 7.4, containing 1 % sucrose and 1 % human serum albumin (HSA). This formulation was defined in order to maintain the biological activity of the protein in a liquid form, stored at −80 °C in the absence of light over 3 years.

### Study design and conduct

The ISS T-003 (ClinicalTrials.gov NCT01513135) was a phase II, randomised, double-blinded, placebo-controlled, clinical trial with the recombinant biologically active HIV-1 B-clade Tat protein conducted at the MeCRU, University of Limpopo, Medunsa Campus (now Sefako Makgatho Health Sciences University), South Africa (Additional file 2: ISS T-003 study protocol). The study was designed to evaluate Tat protein immunogenicity and safety in HIV-1-infected, cART-treated, anti-Tat Ab-negative adult South Africans, and to explore CD4^+^ T-cell numbers and anti-Tat cross-clade neutralizing activity after immunization. The study duration was 48 weeks including an 8-week treatment phase and a 40-week follow-up phase. The allowed window for patients’ screening was 35 days long.

Patients were recruited at the public Health Facilities located in the MeCRU catchment area (Tshwane District). Patients received cART at the Health Facilities throughout the trial. Procedures for patients’ recruitment, access to medical records, referral to the Health Facilities for intervening medical conditions were implemented under the coordination of the South African National Department of Health and the Department of Health of the Gauteng Province, South Africa. A community involvement program was implemented at MeCRU with the support of the South African AIDS Vaccine Initiative, a lead program of the South African   Medical Research Council. MeCRU and local community advisory board and groups implemented community education strategies on HIV/AIDS awareness, participation in clinical trials, recruitment and retention strategies. A Contract Research Organization monitored study conduct, data quality and performed safety data analyses, which were periodically evaluated by the Local Medical Monitor and Data Safety Monitoring Board. The Local Medical Monitor was a blinded sponsor’s representative expert in HIV/AIDS clinical management. He reviewed safety data, assisted the Investigator in assessing adverse events (AEs) severity and causality, and forwarded quarterly reports to the Data Safety Monitoring Board. Data Safety Update Reports were submitted to the Competent Authorities as required.

### Endpoints

The primary endpoint of the study (immunogenicity) was measured by the induction, magnitude and persistence of anti-Tat IgM, IgG and IgA in sera. The secondary endpoint (safety) was assessed by collecting all AEs during the trial, which included vital signs and any clinically significant change in haematological, biochemical and coagulation parameters. All the recorded AEs were classified according to Medical Dictionary for Regulatory Activities (MedDRA) preferred terms and system organ class, and on the basis of drug relationship and grade of severity.

### Study participants

Two hundred adult cART-treated patients were recruited and randomised 1:1 to receive Tat vaccine or placebo. Main criteria for enrolment were the following: age 18–45 years (inclusive), current cART-treatment and chronically suppressed HIV-1 infection as indicated by a HIV-1 plasma viremia <400 copies/mL and a CD4^+^ T-cell count ≥200 cells/µL at screening, and documented at least once during the 12-month period prior to screening irrespective of the pre-cART CD4^+^ nadir, B-clade anti-Tat Ab-negative, willingness and ability to provide informed consent, and no acute illness at study start. Female participants of childbearing potential were required to have a negative pregnancy test at screening and immediately before each vaccination and to use an acceptable method of contraception for at least 3 weeks prior to the first vaccination and for all duration of the trial.

### Study procedures

All participants were randomized to receive the Tat vaccine (30 μg dose) or placebo (vaccine formulation buffer), administered intradermally three times at 4-week intervals (Additional file 2: ISS T-003 study protocol). Randomisation was performed in block sizes of four. Participants were allocated to a randomisation number consisting of a three-digit sequential number pre-fixed by a one-digit unique site identifier. Upon screening completion and immediately prior to vaccine administration, volunteers were randomly assigned to the next available treatment number according to the randomisation schedule, which was generated by the Contract Research Organization using the SAS^®^ procedure PROC PLAN with a randomisation ratio of 1:1. Participants and clinical and laboratory staff, project management personnel and anyone involved in data management or analysis and the sponsor were blinded to treatment assignment. Each investigational product (Tat vaccine/placebo) vial was packaged in one kit-box constituted of three vials with the same label for vaccine or placebo, according to the “Guide to Good Manufacturing Practice for Medicines in South Africa, Version 4.01 March 2009”. Kits were provided to the clinical site in a blinded fashion by the sponsor.

The evaluations performed at each of the 12 study visits varied according to the schedule provided in the Additional file 2: ISS T-003 study protocol . General laboratory assessments, including CD4^+^ T-cell number and HIV plasma viral load were performed by a centralized laboratory (South African National Health Laboratory Service at the Dr. George Mukhari, Ga-Rankuwa, Pretoria). CD4^+^ T-cell counts were performed according to standard national laboratory measurements. HIV-1 viral load was determined with the Abbott Real Time HIV-1 assay (lower limit of detection 40 RNA copies/mL). Blood samples were collected and transferred according to protocol-specific procedures, and tested within 3 h from sample withdrawal. Anti-Tat binding and neutralizing Abs were assessed on cryopreserved specimens shipped by a certified courier to the designated Core Laboratory (Core Laboratory of Immunology and Virology, San Gallicano Institute, Istituti Fisioterapici Ospitalieri, Rome, Italy) according to Standard Operating Procedures.

### Measurement of serum Abs against Tat proteins

The Tat proteins used for anti-Tat Ab determination and for anti-Tat Ab cross-clade analysis were, respectively, from HIV-1 B clade (GenBank accession no.: AAA44199.1); C clade (GenBank accession no.: AAL06113.1); A clade (GenBank accession no.: AAP33775.1); D clade (GenBank accession no.: AAP33758.1) (amino acid sequences are shown in Additional file 1: Figure S1) and were purchased from Diatheva. All proteins were biologically active as determined by the rescue assay with HLM-1 cell line carrying a Tat-defective HIV provirus [19, 20], and/or by Tat uptake by monocyte-derived DC (MDDC) evaluated by intracellular staining for Tat in flow cytometry [35], a potency test that is used to release the Tat vaccine clinical lots.

Serum IgM, IgA and IgG against B-, A-, C-, and D-clade Tat were assessed by enzyme-linked immunosorbent assay (ELISA), as previously described [76]. Briefly, 96-well microplates (Nunc-Immuno Plate MaxiSorp Surface; Nunc) were coated with Tat (100 ng/well) in 200 µL of 0.05 mol/L carbonate-buffer (pH 9.6), and incubated overnight at 4 °C. Wells were washed 5 times with phosphate-buffered solution (PBS), pH 7.4, containing 0.05 % Tween-20, by an automatic plate washer (Asys Hitech flexi wash). Wells were then saturated with PBS containing 1 % bovine serum albumin (BSA) and 0.05 % Tween-20 (Sigma) (blocking buffer) for 90 min at 37 °C and then washed again as above. One hundred microlitres of patient serum samples [diluted at 1:100 (for anti-Tat IgG) or at 1:25 (for anti-Tat IgM or IgA detection) in blocking buffer] were added to the wells and incubated at 37 °C for 90 min. To correct for unspecific binding, each sample was assessed in duplicate against Tat and singly against the buffer in which Tat had been re-suspended. After washing, wells were saturated again with blocking buffer for 15 min at 37 °C, washed again and then a goat anti-human IgG, IgM, or IgA horseradish peroxidase-conjugated secondary Ab (100 µL/well) (PIERCE-Thermo Scientific) was added to each well, and incubated for an additional 90 min at 37 °C. Antigen-bound Abs were revealed by the addition of ABTS [2,2′-azino-bis(3-ethylbenzothiazoline-6-sulphonic acid)] solution (Roche Diagnostics) for 60 min at 37 °C. Absorbance was measured at 405 nm using a microplate reader (BIO-TEK Instruments EL800). The assay was considered valid only when both the positive and negative controls were within ±10 % of variation of the absorbance values recorded in previous 50 assays. For the cut-off calculation, both the optical density (OD) readings at 405 nm of the wells coated with Tat and the delta (Δ) value were utilized. The Δ value was obtained by subtracting the OD reading of the well coated with the buffer alone from the average of the OD values of the two wells coated with the Tat protein. Serum samples were considered positive when both the sample OD at 405 nm and Δ values were ≥0.350 and ≥0.150, respectively. The 0.350 and 0.150 OD values had been previously calculated as three standard deviations (99 % confidence interval) above the mean of each of the absolute and Δ OD values obtained with sera from 89 Italian HIV-negative blood donors and 34 South African HIV-negative individuals. If the sample scored positive, the titer value was 100 for IgG, 25 for IgM and IgA. However, if the OD reading of the sample exceeded both the absolute and Δ OD values by 50 %, serial twofold dilutions of the sample were performed to determine the endpoint titers. Endpoint titers were determined as the reciprocal of the last sample dilution that still had ≥0.350 and ≥0.150 OD values for absolute and Δ parameters. For Tat cross-clade analysis, OD values obtained with the different proteins in the same ELISA test, on the same sample, at the same dilution, were compared.

### Measurement of serum Abs against the Env protein

The same ELISA protocol and criteria for cut-off determination were applied for measurement of anti-Env Abs and their titer definition [70]. The ΔV2-Env (Novartis Vaccine and Diagnostics) from the HIV-1 C-clade TV1 strain was used. Only IgG Abs were tested, starting from a 1:100 dilution.

### Anti-Tat neutralizing Ab responses

Anti-Tat neutralizing activity in sera was assessed by Tat-mediated Env entry in DC as described [35]. Briefly, MDDC from blood of healthy donors were cultured and induced to maturation as described [35, 36]. Purity of MDDC was always ≥99 %. Sera were diluted 1:30 in PBS and incubated for 60 min at 37 °C with B- or C-clade trimeric Env (0.4 μM in monomer) (Novartis) previously mixed for 10 min at 25 °C with B- or C-clade Tat (0.4 μM) or degassed PBS (control). Samples were then added to MDDC (2 × 10^5^ cells/mL) to a 1:5 final dilution and incubated for 10 min at 37 °C. Cells were then washed with cold medium and treated for 10 min at 37 °C with ethylene diamine tetra-acetic acid (EDTA) (Life Technologies) to remove any externally bound protein. After fixation and permeabilization, DC were stained with rabbit anti-gp120 polyclonal Abs (Chem Progress) or purified rabbit-IgG control Abs (Sigma-Aldrich), followed by fluorescein isothiocyanate (FITC)-conjugated anti-rabbit Ig (Pierce). Fluorescence was measured by flow cytometry and results expressed as the percentage of Env-positive cells as compared to isotype-stained samples. Sera were defined as “neutralizing” when capable of inhibiting Env entry into DC in the presence of Tat by at least 50 % as compared to baseline sera values (ND_50_).

### Sample size calculation

Sample size for this study was powered for immunogenicity evaluation. The immunogenicity was assumed to be 80 % for vaccinees and 60 % for placebos, with alpha set at 0.05 (two-tailed). According to this assumption, a sample size of 91 per group had 80 % power of showing statistical significance (p < 0.05). The assumed response rate for vaccinees was based on the results of studies conducted at the time of protocol preparation. The response rate in placebos was set in the absence of reference-controlled studies and was therefore very conservative. The actual power of the study, given the percentage of spontaneous seroconversion, is 99 %.

### Statistics

Two populations were considered for statistical analyses: the immunogenicity population (199 subjects), representing all randomised individuals who received at least two immunizations, and the safety population (200 subjects), representing all randomised subjects who received at least one immunization. Subjects with at least one positive anti-Tat Ab response at any given time point during the study were defined as “responders”. Ninety-five percentage confidence intervals were estimated for the primary endpoints; comparison between treatment groups was performed using the Chi square test. Kaplan–Meier method was used to assess the cumulative probability of anti-Tat Ab persistence, by treatment groups, and compared by the log-rank test. Anti-Tat Ab titers and the percentage of DC internalizing Env were compared between vaccinees and placebos by the Student’s t test after log_10_ transformation to normalize the data distribution. Wilcoxon signed-rank test was used to assess the intensity of cross-clade anti-Tat Abs (measured as OD units) after immunization. Longitudinal analysis for repeated measures was applied for analysis of CD4^+^ T-cell number, after controlling normality assumption of variable distribution (Saphiro–Wilk test). The relationship between Tat-mediated Env entry in DC and anti-Tat or anti-Env Ab-binding titers or CD4^+^ T-cell number was assessed by the longitudinal regression model using the generalized estimating equations method. Wilcoxon signed-rank test was used to assess changes from baseline of CD4^+^ T-cell number in subjects not compliant to cART, while Wilcoxon–Mann–Whitney test was performed in order to evaluate differences between non-cART-compliant vaccinees and placebos at each visit. Statistical analyses were carried out at two-sided with a 0.05 significance level, using SAS^®^ (Version 9.2, SAS Institute Inc., Cary, NC, USA).

## Results

### Patients accrual and demographic data

**Fig. 1:**
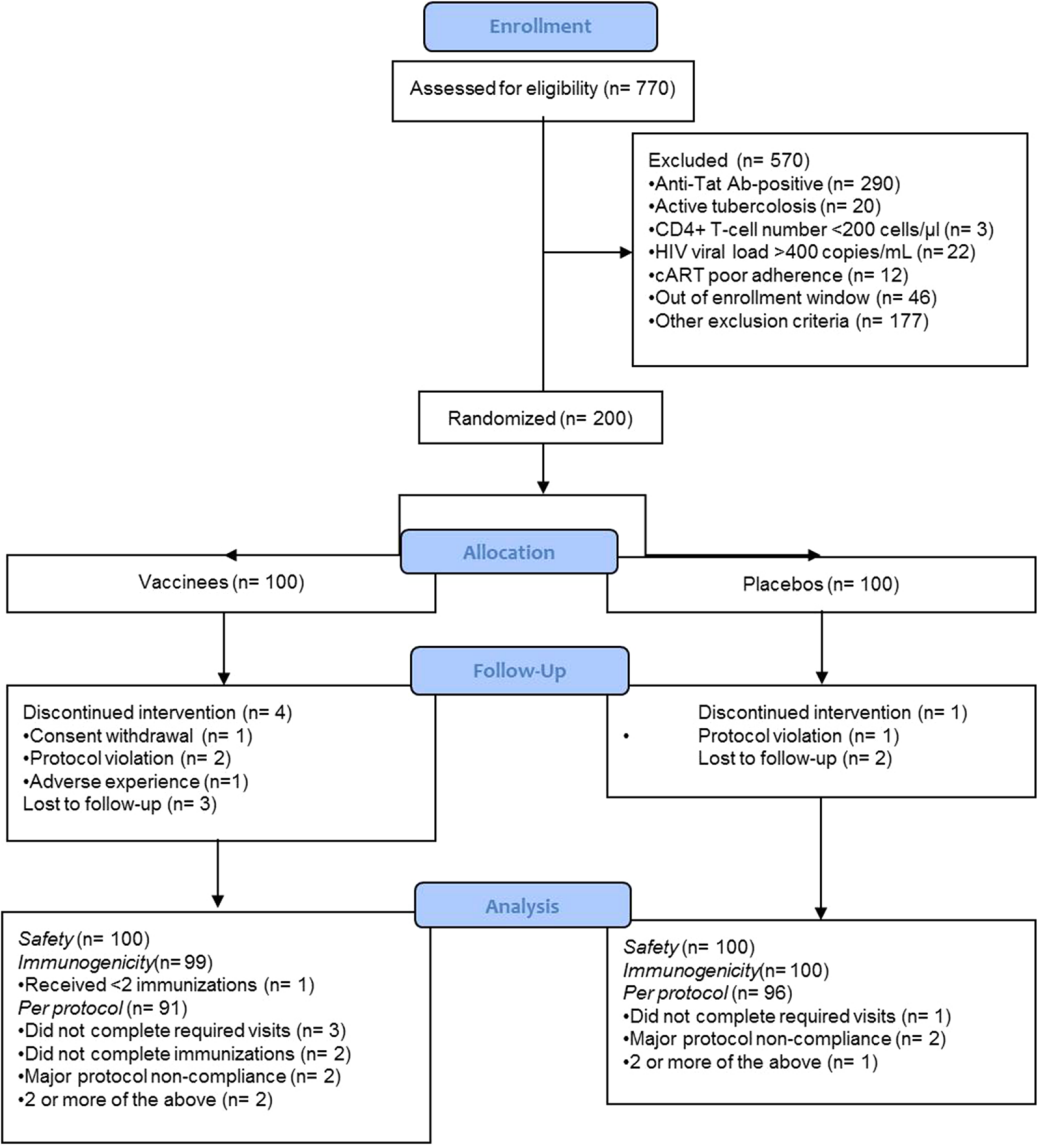
CONSORT flow diagram. The number of participants screened, enrolled, randomized, followed-up and analyzed is shown for vaccine and placebo groups. Two hundred participants were randomised to one of the two treatment groups and analyzed for safety (safety population). One subject who received only one immunization was excluded from the immunogenicity population (total = 199). Thirteen volunteers were excluded from the “Per protocol” analysis: four received <3 immunizations, four did not complete three or more visits of follow-up and five had major protocol non-compliance (total = 187)

Seven-hundred-seventy cART-treated patients were assessed for eligibility (Fig. 1). Two hundred participants were enrolled between February 27, 2012 and 13 June, 2013. Study was completed in June 2014. Participants were randomised 1:1 to one of the two treatment groups. Ninety-seven percent of enrolled participants completed the study.

All individuals were analyzed for safety. One subject who received only one immunization was excluded from the immunogenicity population (Fig. 1). Thirteen volunteers were excluded from the “Per Protocol” analysis for major protocol non-compliance (Fig. 1). Baseline demographic and clinical characteristics of participants are shown in Table 1. Twenty-six percent of participants were male and 74 % female; all were black, except one volunteer. At the study entry the mean age was 36 years for both vaccinees and placebos. In vaccinees, the mean CD4^+^ T-cell count was 510 cells/µL, 95 % of them had undetectable HIV RNA, the mean years from HIV diagnosis was 5.0, while the mean time on cART was 3.5 years, with 97 % on non-nucleoside reverse-transcriptase inhibitors (NNRTI) or nucleoside reverse transcriptase inhibitors (NRTI)-based and 3 % on PI-based regimens. In placebos, the mean CD4^+^ T-cell count was 563 cells/µL, HIV RNA was undetectable in 96 % of them, the mean years from HIV diagnosis was 4.9 years, while the mean time on cART was 3.3, with 98 % on NNRTI or NRTI-based and 2 % on PI-based regimens.

**Table 1:** Baseline characteristics of study participants

	n	Vaccinees	n	Placebo
Gender				
Male	32	32.0 %	20	20.0 %
Female	68	68.0 %	80	80.0 %
Race				
Black	100	100.0 %	99	99.0 %
Caucasian	0	0.0 %	0	0.0 %
Mixed	0	0.0 %	1	1.0 %
Age				
Mean ± SD	100	36.1 ± 5.6	100	36.0 ± 6.2
Range		21.1–45.8		19.6–45.4
CD4^+^ (cells/μL)				
Mean ± SD	99	510 ± 229	100	563 ± 195
Range		137–1530		242–1252
CD4^+^ (%)				
Mean ± SD	99	28 ± 8	100	29 ± 7
Range		7–49		17–42
HIV RNA (copies/mL)				
<40 (assay cut-off)	94	95.0 %	96	96.0 %
≥40	5	5.0 %	4	4.0 %
Years from HIV diagnosis				
Mean ± SD	100	5.0 ± 3.0	100	4.9 ± 3.3
Range		1.0–14.0		1.0–19.0
Years from cART initiation				
Mean ± SD	100	3.5 ± 2.0	100	3.3 ± 2.1
Range		0.7–8.2		0.6–8.9
cART regimen				
NNRTI or NRTI-based	97	97.0 %	98	98.0 %
PI-based	3	3.0 %	2	2.0 %
Previous tuberculosis	29	29.0 %	34	34.0 %

*n* number of individuals, *SD* standard deviation

### HIV-1 B-clade Tat vaccine safety and tolerability

Tat immunization was safe and well tolerated without relevant differences between vaccinees and placebos. In particular, 190 patients (96 vaccinees and 94 placebos) experienced at least one AE during the study, mainly of mild intensity (Table 2). General disorders and administration site conditions were the most frequent AEs related to study treatment both in vaccinees (73 %) and placebos (58 %), followed by nervous system disorders (mainly headache events), which had higher incidence in placebos (38 %) than vaccinees (27 %) (Table 3). No serious AEs (SAE) related to study treatment or suspected unexpected adverse reactions were reported. The non-serious AEs related to study drug were mostly mild and local. Most clinically relevant abnormal laboratory events were reported with a similar frequency in both the treatment groups and were considered unrelated, since they are findings typically associated with HIV-1 infection (i.e. low haemoglobin, low neutrophil and white cell counts, increased viral load). Eight participants (two placebos and six vaccinees) reported at least one SAE (unrelated to study treatment). In particular, one placebo underwent hysterectomy and one was diagnosed with type-II diabetes mellitus. Among the vaccinees, two participants were diagnosed with pulmonary tuberculosis, one patient was admitted to the hospital for respiratory tract infection, bronchiectasis-empyema thoracis and abdominal pain, one patient underwent hysterectomy, and intentional self-injury was reported in two participants. All these SAE resolved completely, except the type II diabetes mellitus.

**Table 2:** Total adverse events observed in study participants reported by relationship to study drug and intensity

	Treatment group								
	Tat vaccine			Placebo			Total		
	n	(m)	%	n	(m)	%	n	(m)	%
Number of subjects in safety population	100			100			200		
Number of subjects with at least one adverse event	96	(883)	96.0	94	(581)	94.0	190	(1464)	95.0
Relationship with study medication									
Certain	72	(541)	72.0	59	(250)	59.0	131	(791)	65.5
Probable	14	(35)	14.0	23	(38)	23.0	37	(73)	18.5
Possible	25	(45)	25.0	28	(56)	28.0	53	(101)	26.5
Unlikely	41	(80)	41.0	37	(63)	37.0	78	(143)	39.0
Not related	76	(182)	76.0	74	(174)	74.0	150	(356)	75.0
Not assessable	0	(0)		0	(0)		0	(0)	
Not known	0	(0)		0	(0)		0	(0)	
Intensity									
Mild	94	(774)	94.0	91	(522)	91.0	185	(1296)	92.5
Moderate	42	(90)	42.0	28	(48)	28.0	70	(138)	35.0
Severe	15	(17)	15.0	9	(10)	9.0	24	(27)	12.0
Not applicable	1	(1)	1.0	0	(0)		1	(1)	0.5
Not known	1	(1)	1.0	1	(1)	1.0	2	(2)	1.0
Serious adverse events	6	(8)	6.0	2	(2)	2.0	8	(10)	4.0
Related	0	(0)	0.0	0	(0)	0.0	0	(0)	0.0
Not related	6	(8)	6.0	2	(2)	2.0	8	(10)	4.0

n = number of subjects, (m) = number of mentions, % = all percentages are expressed as the percentage of the number of subjects in the safety population in each treatment group

**Table 3:** Incidence of related adverse events by system organ class and relationship to study treatment

MedDRA system organ class	Treatment group														
	Tat vaccine						Placebo						Total		
	Related^a^			Not related^b^			Related^a^			Not related^b^					
	n	(m)	%	n	(m)	%	n	(m)	%	n	(m)	%	n	(m)	%
Number of subjects	100			100			100			100			200		
Number of subjects with at least one adverse event	77	(621)	77.0	85	(262)	85.0	72	(344)	72.0	81	(237)	81.0	190	(1464)	95.0
General disorders and administration site conditions	73	(520)	73.0	8	(10)	8.0	58	(199)	58.0	11	(12)	11.0	133	(741)	66.5
Infections and infestations	1	(1)	1.0	50	(77)	50.0	2	(2)	2.0	56	(88)	56.0	107	(168)	53.5
Nervous system disorders	27	(40)	27.0	19	(24)	19.0	38	(67)	38.0	10	(13)	10.0	81	(144)	40.5
Musculoskeletal and connective tissue disorders	16	(25)	16.0	14	(16)	14.0	17	(31)	17.0	15	(28)	15.0	54	(100)	27.0
Gastrointestinal disorders	9	(12)	9.0	20	(25)	20.0	14	(18)	14.0	13	(19)	13.0	49	(74)	24.5
Skin and subcutaneous tissue disorders	10	(12)	10.0	15	(15)	15.0	10	(18)	10.0	13	(16)	13.0	47	(61)	23.5
Reproductive system and breast disorders	0	(0)		22	(24)	22.0	0	(0)		22	(27)	22.0	44	(51)	22.0
Investigations	2	(3)	2.0	19	(28)	19.0	2	(2)	2.0	8	(10)	8.0	30	(43)	15.0
Blood and lymphatic system disorders	7	(7)	7.0	7	(9)	7.0	5	(6)	5.0	3	(3)	3.0	21	(25)	10.5
Injury, poisoning and procedural complications	0	(0)		10	(12)	10.0	0	(0)		5	(7)	5.0	15	(19)	7.5
Vascular disorders	1	(1)	1.0	6	(6)	6.0	0	(0)		3	(3)	3.0	10	(10)	5.0
Respiratory, thoracic and mediastinal disorders	0	(0)		2	(2)	2.0	0	(0)		4	(5)	4.0	6	(7)	3.0
Eye disorders	0	(0)		3	(3)	3.0	0	(0)		2	(2)	2.0	5	(5)	2.5
Metabolism and nutrition disorders	0	(0)		3	(3)	3.0	0	(0)		1	(1)	1.0	4	(4)	2.0
Renal and urinary disorders	0	(0)		2	(2)	2.0	0	(0)		1	(1)	1.0	3	(3)	1.5
Surgical and medical procedures	0	(0)		2	(2)	2.0	0	(0)		1	(1)	1.0	3	(3)	1.5
Psychiatric disorders	0	(0)		2	(4)	2.0	0	(0)		0	(0)		2	(4)	1.0
Cardiac disorders	0	(0)		0	(0)		1	(1)	1.0	0	(0)		1	(1)	0.5
Immune system disorders	0	(0)		0	(0)		0	(0)		1	(1)	1.0	1	(1)	0.5

n = number of subjects, (m) = number of mentions, % = all percentages are expressed as the percentage of subjects in the safety population in each treatment group. Adverse event data were coded using the MedDRA dictionary version 15.0

^a^Related refers to events whose relationship to the study treatment was regarded as certain, probable or possible

^b^Not related refers to events whose relationship to the study treatment was regarded as unrelated or unlikely related

Since no “important safety events” or “significant findings” emerged during the study, the Data Safety Monitoring Board concluded that the Tat vaccine is safe and well tolerated.

### HIV-1 B-clade Tat vaccine induces durable anti-Tat Abs of all subclasses

**Fig. 2:**
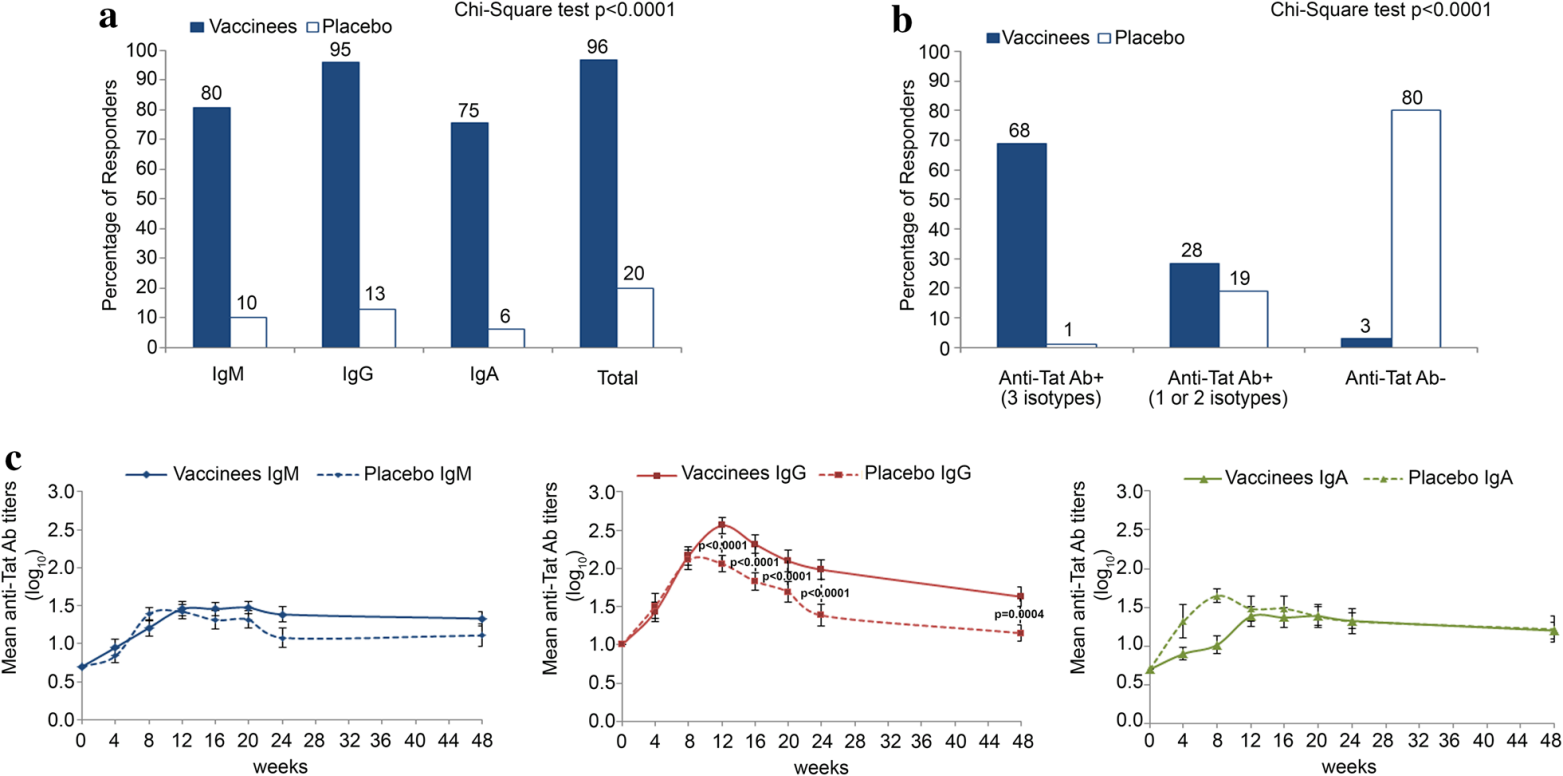
Anti-Tat humoral immune response elicited in study participants. **a** Percentage of responders for anti-Tat Abs (see “Methods” section) in vaccinees (n = 99) or placebos (n = 100). The absolute number of vaccines/placebos developing anti-Tat Ig subclasses are reported on the *top* of each histogram. Statistical significant differences were detected between vaccinees and placebos for each Ig and for total response (p < 0.0001, Chi square test). **b** Percentage of responders for anti-Tat Abs stratified according to the presence of one or more Ab isotype in vaccinees (n = 99) or placebos (n = 100). The absolute number of vaccines/placebos developing one or more Ab isotype are reported on the *top* of each histogram. Statistical significant differences were detected between vaccinees and placebos (p < 0.0001, Chi square test). **c** IgM, IgG and IgA Ab mean titers (with standard error) in responders (vaccinees: n = 79 for IgM, n = 95 for IgG and n = 75 for IgA; placebos: n = 9 for IgM, n = 12 for IgG and n = 6 for IgA). Significant differences were detected between vaccinees and placebos for anti-Tat IgG Abs from week 12 to week 48 (Student’s t test)

**Fig. 3:**
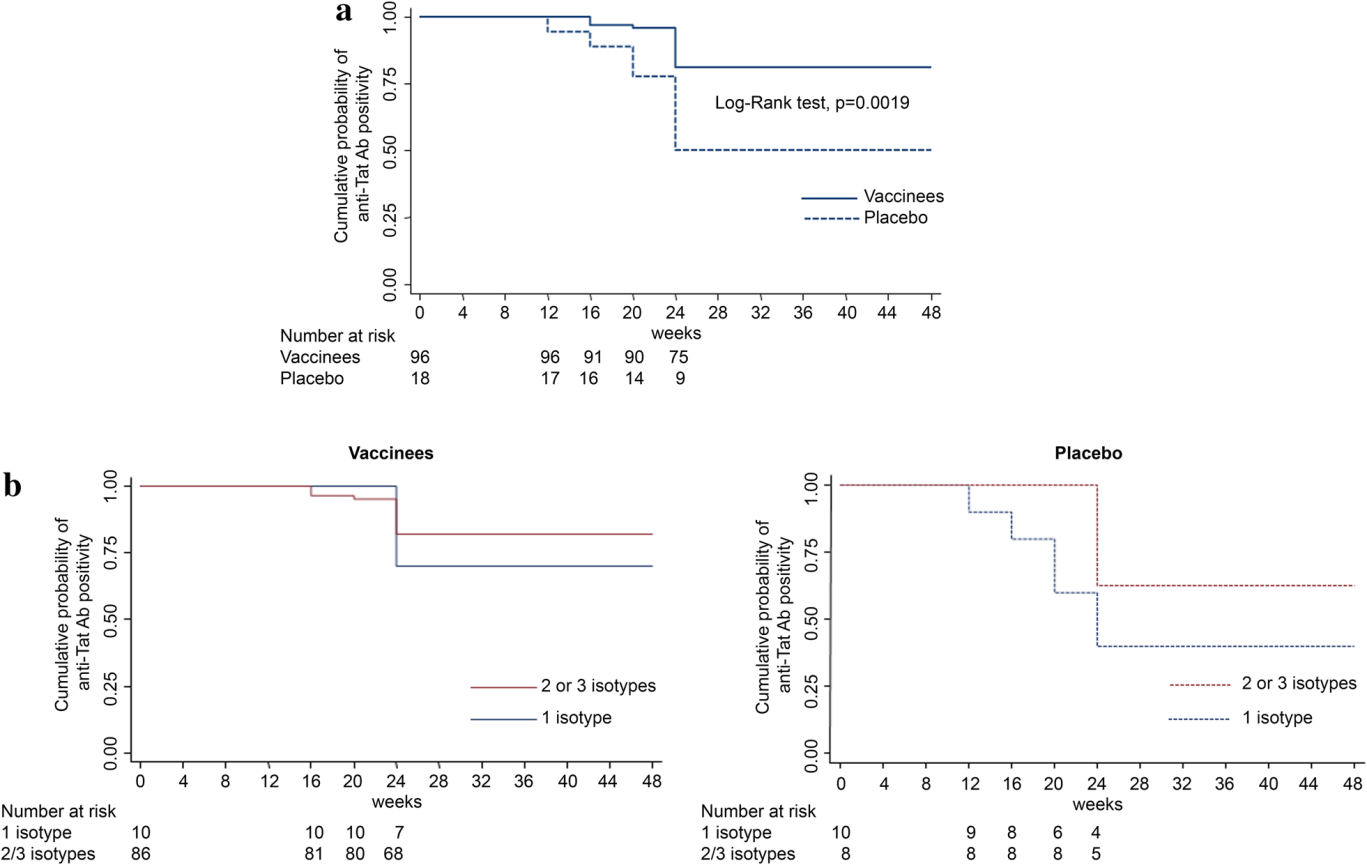
Anti-Tat Ab durability in responders. **a** Kaplan–Meier estimates showing the cumulative probability of anti-Tat Ab durability during follow-up in responders (see “Methods” section) (vaccinees: n = 96; placebos: n = 18). Anti-Tat Abs persisted significantly longer in vaccinees as compared to the placebo group (p = 0.0019, log-rank test). **b** Kaplan–Meier estimates showing the cumulative probability of anti-Tat Ab durability during follow-up in vaccinees (*left panel*) or placebo (*right panel*) responders, according to the number of anti-Tat Ab isotypes (vaccinees: one subclass n = 10, two or three subclasses n = 86; placebo: one subclass n = 10, two or three subclasses n = 8)

Tat immunization induced anti-Tat B-clade Abs in 97 % of vaccinees, whereas 20 % of placebos developed spontaneously anti-Tat Abs (all immunogenicity population evaluated). Anti-Tat Ab responses detected in vaccinees and placebos were significantly different (Chi square test, p < 0.0001, both for total Abs and Ig subclasses). In particular, 81 % of vaccinees developed anti-Tat B-clade IgM, 96 % IgG, and 76 % IgA, as opposed to 10 % IgM, 13 % IgG, and 6 % IgA of placebos, respectively (Fig. 2a). As shown in Table 4 and Fig. 2b, 69 % of vaccinees developed anti-Tat Abs of all Ig subclasses, 28 % developed one or two Ig subclasses, and 3 % of vaccinees had no detectable anti-Tat Abs. In contrast, 1 % of placebos developed anti-Tat Abs of all three Ig subclasses, 19 % of one or two subclasses, and 80 % had no detectable anti-Tat Abs (Chi square test, p < 0.0001, Fig. 2b). Anti-Tat B-clade Ab mean titers peaked between week 8 and week 12 for all Ig subclasses and statistically significant differences for IgG titers between vaccinees and placebos were observed since week 12 (weeks 12–24, p < 0.0001; week 48 p = 0.0004) (Fig. 2c). Moreover, anti-Tat Ab responses persisted significantly longer in vaccinees as compared to placebos (log-rank test, p = 0.0019) (Fig. 3a). Further, Ab persistence was longer in vaccinees and placebos with 2 or 3 Ab subclasses as compared to those with 1 subclass (Fig. 3b, c). The “Per Protocol” analysis confirmed the results from the immunogenicity population, in particular, 88/91 (97 %) and 20/96 (21 %) of vaccinees and placebos, respectively, developed anti-Tat Abs (p < 0.0001).

**Table 4:** Anti-Tat Ab response by Ig subclasses in vaccinees and placebos

	Vaccinees (*n* = 99)		Placebos (*n* = 100)	
	n	%	n	%
IgM+	1	1.0	5	5.0
IgG+	9	9.1	5	5.0
IgA+	0	0.0	2	2.0
IgM + IgG+	11	11.1	4	4.0
IgM + IgA+	0	0.0	0	0.0
IgG + IgA+	7	7.1	3	3.0
IgM + IgG + IgA+	68	68.7	1	1.0
Ab-negative	3	3.0	80	80.0

Percentage of subjects positive for 1, 2 or 3 anti-Tat Ab subclasses at any given time point after the first immunization

*n* number of subjects

### Vaccination with the HIV-1 B-clade Tat protein elicits Abs also recognizing Tat from A, C and D clades

The presence of anti-Tat Abs against clades other than B (i.e. C, D, A) was evaluated in the 99 vaccinees. Fifty-one patients that were negative at baseline also for Abs against Tat from A, C, or D clade, after immunization with the B-clade Tat protein developed anti-Tat Abs recognizing Tat from one or more of these other clades (Table 5), in addition to Tat B clade.

**Table 5:** Induction of anti-Tat cross-clade Abs after immunization in vaccinees negative at baseline for any anti-Tat Abs

HIV clades	n	%
C	5	9.8
D	7	13.7
A	4	7.8
C + D	12	23.5
C + A	1	2.0
D + A	7	13.7
C + D + A	15	29.4
Total	51	100.0

Sera from 51 vaccinees negative at baseline also for anti-Tat Abs against C, D and A clades were tested between week 12 and week 24 after immunization with the B-clade Tat protein. All patients mounted anti-Tat Ab responses against A, C, and/or D clade

**Fig. 4:**
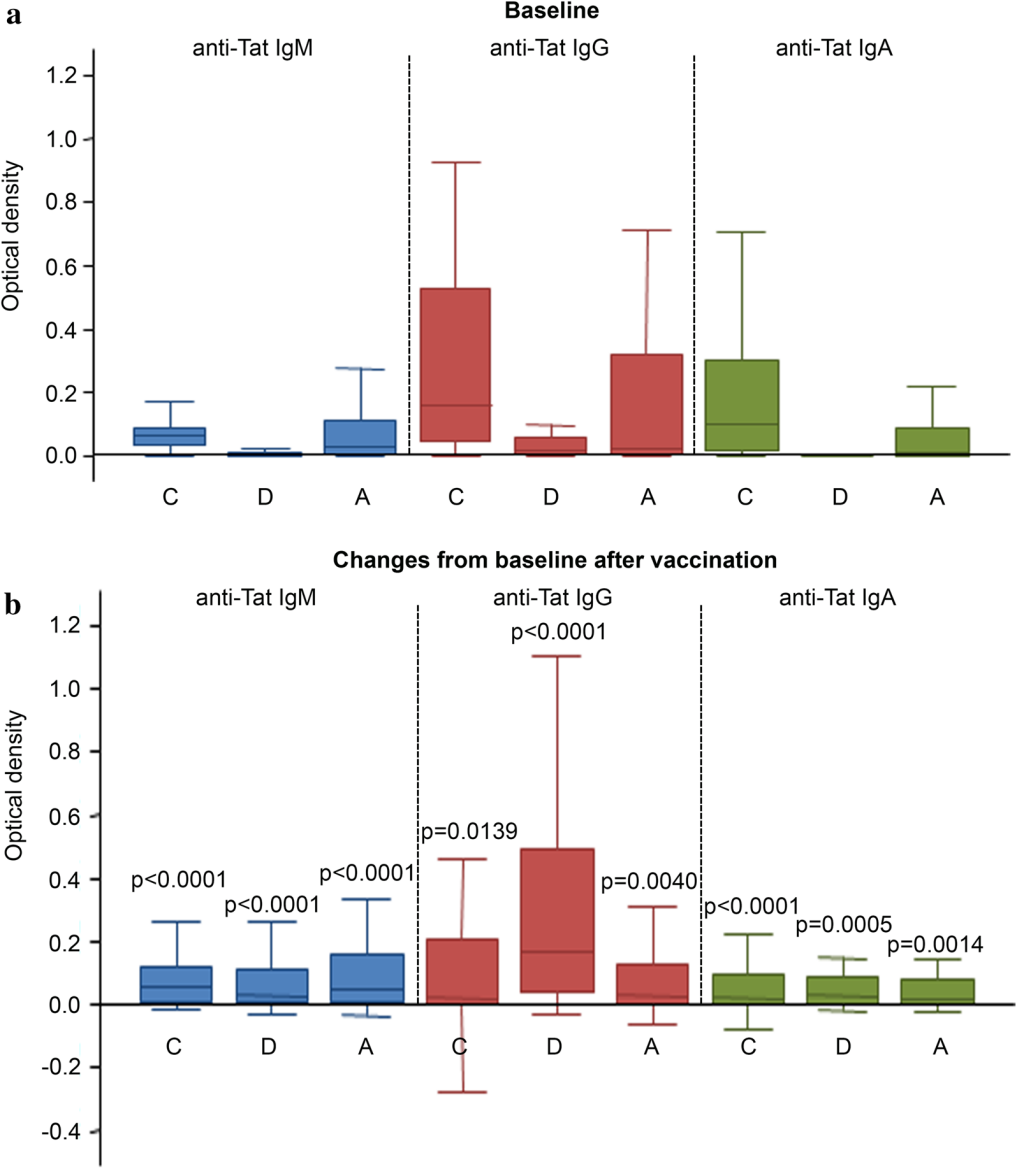
Increase of cross-clades anti-Tat Abs elicited in vaccinees. **a** Baseline OD values of anti-Tat IgM, IgG and IgA against clades *C*, *D* and *A* in vaccinees prior to immunization (n = 29, 76 % *C* clade, 41 % A clade, 14 % *D* clade). **b** Changes from baseline of IgM, IgG and IgA Ab responses (OD) against Tat from other clades (*C*, *D*, *A*) after vaccination. Testing was performed at the peak of Ab responses (between 12 and 24 weeks). Statistical analysis was performed using the Wilcoxon signed-rank test. p values assess the increase from baseline

At baseline (Fig. 4a), 29 vaccinees, although negative for anti-Tat Abs against B-clade Tat, had Abs against Tat of one or more of the other clades tested (76 % C clade, 41 % A clade, 14 % D clade). After vaccination, all of them experienced a statistically significant increase of intensity of these responses (Fig. 4b). In particular, changes of intensity from baseline levels were similar for IgM and IgA for all clades, while for IgG changes were higher for C and D clades.

### Vaccination with the HIV-1 B-clade Tat protein induces cross-clade neutralizing anti-Tat Abs

**Fig. 5:**
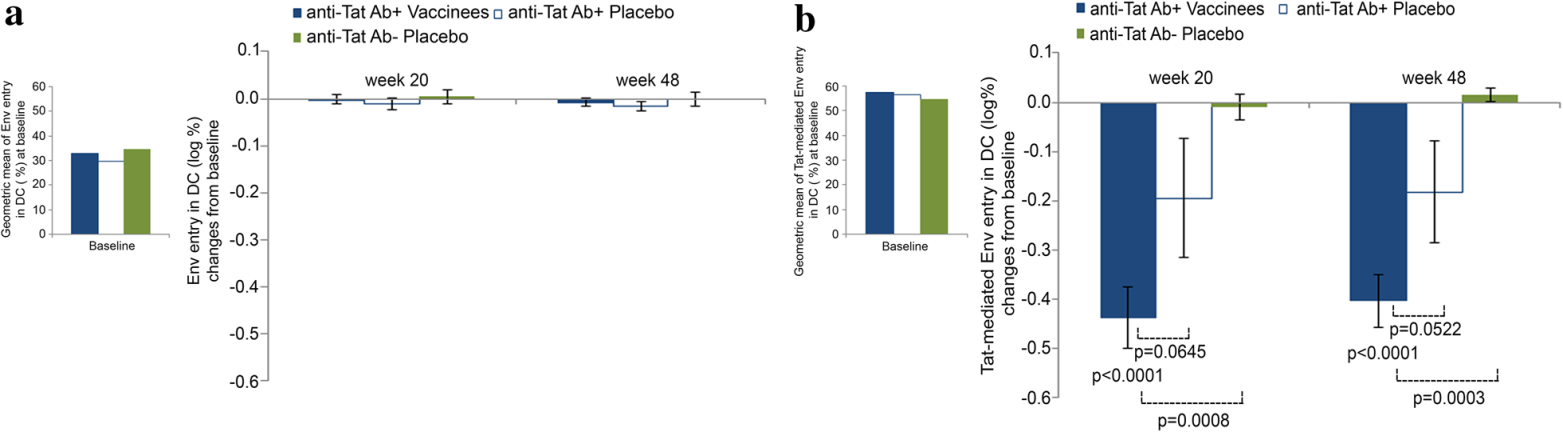
Neutralization of Tat/Env complex entry in DC. Baseline values (*left panels*) and changes from baseline after immunization (*right panels*) of B-clade Env entry in DC in the absence (**a**) or presence (**b**) of B-clade Tat in anti-Tat Ab-positive (n = 13) vaccinees, and anti-Tat Ab-positive (n = 6) or anti-Tat Ab-negative (n = 5) placebos at week 20 and 48 from the first immunization. Reduction of Env entry in DC by sera indicates neutralization. Student’s t test was applied to evaluate the changes from baseline within and between treatment groups

**Fig. 6:**
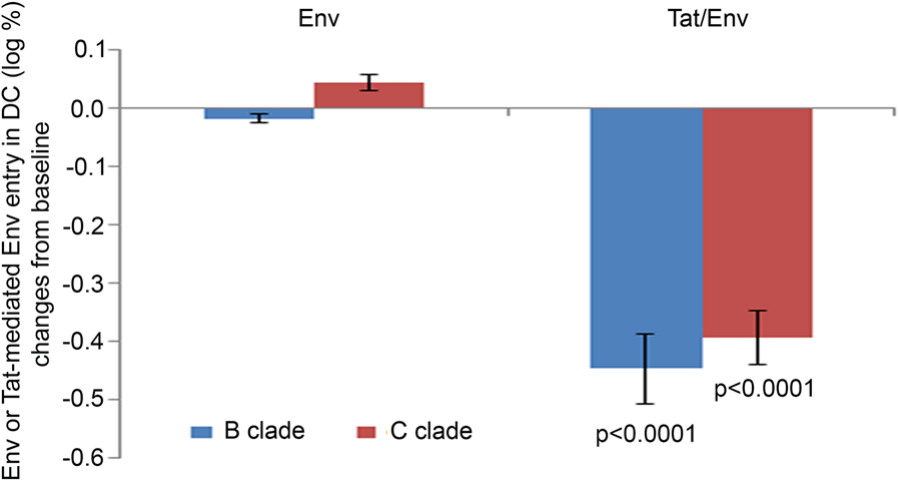
Neutralization of B- and C-clade Tat/Env complex entry in DC in vaccinees. Neutralization of B- (n = 13) and C- (n = 10) clade Env entry in DC in the presence or absence of (B- or C-clade) Tat by sera of Ab-positive vaccinees, measured at week 20 or week 48 after immunization. Data are presented as mean values with standard errors. Student’s t test for paired data was used for the analyses

The neutralization of B-clade Tat-mediated entry of oligomeric B-clade Env in DC was used to investigate anti-Tat Ab functional activity in 24 participants. This assay permits measuring HIV neutralization even in the presence of cART [57, 65], which interferes with traditional infection assays [77]. At baseline, entry of Env in the absence of Tat was comparable for all sera (Fig. 5a, left panel) and did not change after immunization (Fig. 5a, right panel). As shown previously [57], Tat increased entry of Env with all sera prior to immunization (baseline) (Fig. 5b, left panel), whereas sera from vaccinees strongly reduced Env entry (more than 60 %) (week 20 and 48, p < 0.0001). This occurred to a lesser extent also with sera from anti-Tat Ab-positive placebos (about 37 % reduction) (Fig. 5b, right panel). No changes were observed in Ab-negative placebos (Fig. 5b, right panel). Differences between vaccinees and anti-Tat Ab-negative placebos were statistically significant at both time points examined (week 20 and week 48 p = 0.0009 and p = 0.0003, respectively). Further, differences in reaching 50 % neutralization (ND_50_) of Env entry were observed between vaccinees (11/13, 85 %) and the anti-Tat Ab-positive placebos (2/6, 33 %) (Fisher’s exact test, p = 0.0460). Neutralization of Tat-mediated Env entry in DC was also analyzed for C clade Tat and Env. As shown in Fig. 6, anti-Tat Abs elicited by vaccination with the B-clade Tat protein induced cross-clade neutralizing Abs against B and C clade Tat/Env complex entry in DC (p < 0.0001 for both clades).

### Anti-Tat but not anti-Env Abs correlate with neutralization of Env entry in vaccinees

To evaluate the role of both anti-Tat and anti-Env humoral responses on the neutralization of Tat-mediated entry of oligomeric Env in DC, anti-Env Abs were also tested (all immunogenicity population evaluated). At baseline, all subjects had anti-Env Abs (geometric mean Ab-titers 72,408, range 200–409,600) with titers that did not change significantly during the follow-up (data not shown) and correlated positively with the levels of Tat-mediated Env entry in DC (r = 0.42, p = 0.0214), indicating lack of neutralization. In contrast, after immunization, vaccinees showed a significant inverse relationship between anti-Tat IgM or IgG Ab titers (p = 0.0853 and p = 0.0039, respectively) or anti-Env IgG titers (p = 0.0015) and the levels of Tat-mediated Env entry in DC (Table 6), indicating correlation with neutralization of Env entry. Of note, anti-Env Ab titers did not correlate with neutralization of Env entry in anti-Tat Ab-negative placebos, indicating that anti-Env Abs require anti-Tat Abs to inhibit the Tat/Env complex formation and virus entry, as shown earlier both in vitro and in vivo [65, 70, 78].

**Table 6:** Relationship between anti-Tat or anti-Env Ab titers and Tat-mediated Env entry in DC in vaccinees

Parameter	Estimate	95 % CI		p value
Vaccinees				
Anti-Tat IgM (log_10_ titers)	−0.15	−0.31	0.02	0.0853
Anti-Tat IgG (log_10_ titers)	−0.12	−0.20	−0.04	0.0039
Anti-Tat IgA (log_10_ titers)	−0.02	−0.12	0.08	0.7579
Anti-Env IgG (log_10_ titers)	−0.06	−0.09	−0.02	0.0015
Placebos				
Anti-Env IgG (log_10_ titers)	0.00	−0.02	0.02	0.9471

A longitudinal analysis for repeated measures by generalized estimating equation method was used for the analysis. Vaccinees anti-Tat Ab-positive n = 19 (86 observations), placebos anti-Tat Ab-negative n = 5 (30 observations)

*CI* confidence interval

### Tat vaccination induces CD4^+^ T cell number increases, which correlate with neutralization

**Fig. 7:**
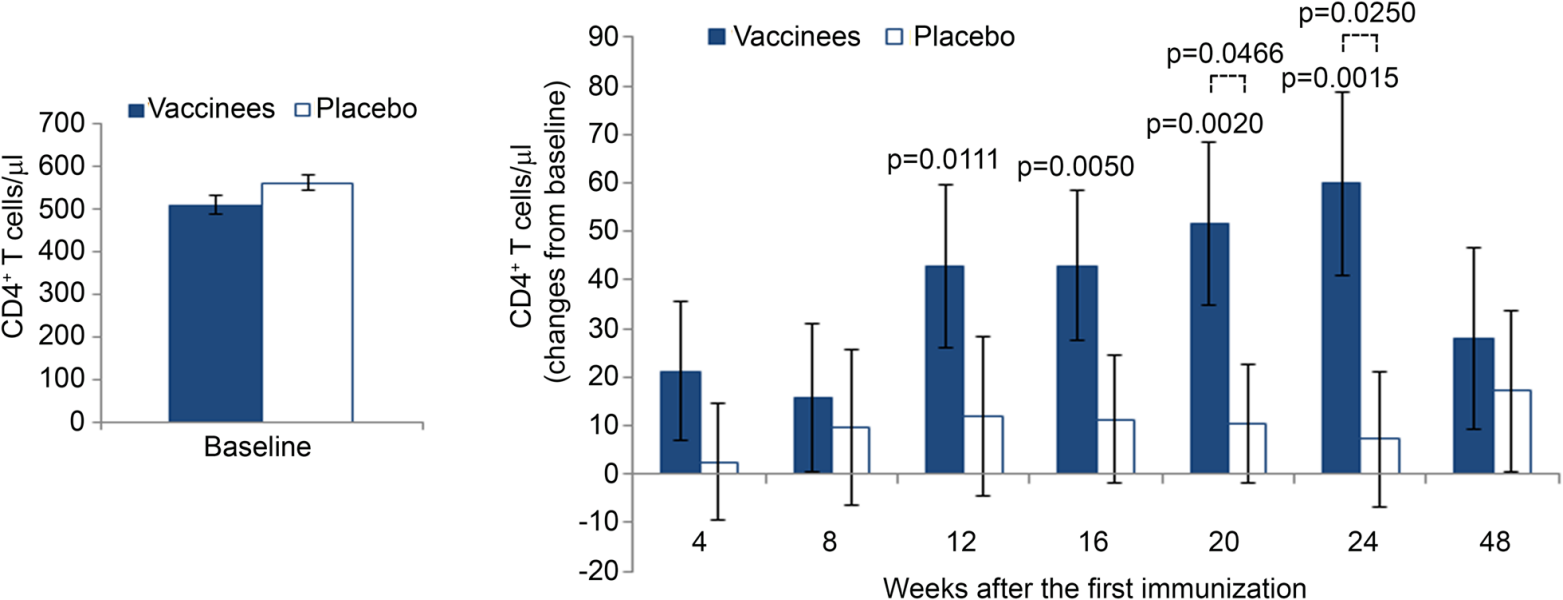
Changes from baseline of CD4^+^ T-cell number in vaccinees and placebos. Baseline values (*left panel*) and changes from baseline (*right panel*) of CD4^+^ T-cell counts in vaccinees (n = 99) and placebos (n = 100). Data are presented as mean values with standard errors. Longitudinal analysis for repeated measures by the generalized estimating equations method was applied for the analysis. p values assess the changes from baseline within and between treatment groups

Compared to placebos, CD4^+^ T-cell counts increased significantly and progressively in vaccinees (Fig. 7) up to week 24 when they peaked (mean gain of 60 cells/µL p = 0.0015), whereas at the end of the study (week 48) the mean gain compared to baseline values was of 28 cells/µL. In contrast, placebos showed a slower kinetics and lower, and not statistically significant, increases (mean gain of 11 cells/µL), which peaked at 48 weeks with a mean value of 17 cells/µL as compared to baseline. CD4^+^ T-cell counts were also analyzed by treatment groups over time by applying a random-effect regression model. The increase from baseline of CD4^+^ T cells up to week 24 was 2.2 cells/µL (95 % CI 1.1; 3.2, p < 0.0001) per week in vaccinees and 0.1 cells/µL (95 % CI 0.7; 3.4) per week in the placebo group, respectively. The difference between the coefficients of regression was statistically significant (p = 0.0031). The comparison between the two arms showed statistically significant changes from baseline at week 20 (p = 0.0466) and week 24 (p = 0.0250).

**Fig. 8:**
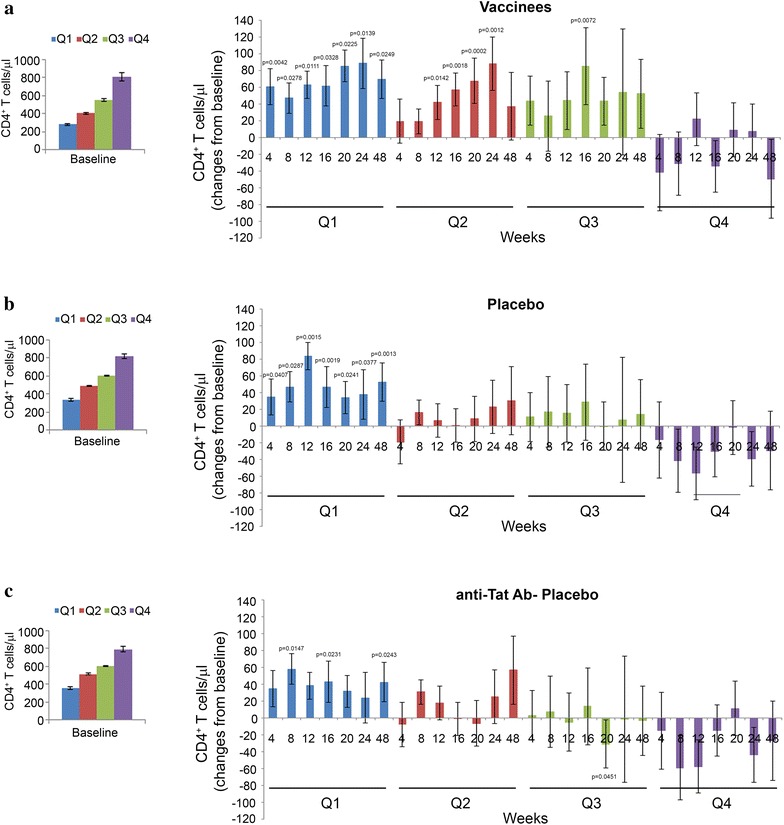
CD4^+^ T-cell numbers up to week 48 in vaccinees and placebo stratified by quartiles according to baseline values. Baseline values (*left panels*) and changes from baseline (*right panels*) of CD4^+^ T cells in **a** vaccinees (n = 98), **b** placebo (n = 100) and **c** anti-Tat Ab-negative placebo (n = 80). Data are presented as mean values with standard errors. Longitudinal analysis for repeated measures was used. p values assess the changes from baseline within each treatment group

To evaluate the effect of vaccination on the increase of CD4^+^ T cells according to their levels at study entry, baseline values were stratified by quartiles. Increases up to about 90 cells/µL were detected in vaccinated subjects in Q1, Q2, and Q3, while no significant changes were observed in Q4, indicating that vaccination had major effects in subjects with lower CD4^+^ T cell number at baseline (Fig. 8). Placebos showed significant CD4^+^ T-cell increases only in Q1 (up to 84 cells/µL). Of note, the gaining in CD4^+^ T-cell counts in Q1 was lower (up to 58 cells/µL) in placebos negative for anti-Tat Abs, who also experienced a significant CD4^+^ T-cell decay in Q3 at week 20 (Fig. 8).

Further, CD4^+^ T-cell increases correlated significantly with neutralization of Env entry in DC in vaccinees (n = 19) (p = 0.0023) as compared to placebos (n = 11) (Table 7).

**Table 7:** Longitudinal analysis of Tat-mediated Env entry in DC versus CD4^+^ T-cell counts

Treatment	Estimate	95 % CI		p value
Vaccinees	−127	−208	−45	0.0023
Placebo	−72	−194	51	0.2515

A significant inverse relationship was observed between CD4^+^ T cells and the Tat-mediated Env entry in DC in the presence of sera from vaccinees (n = 19) but not from placebo (n = 11) indicating a positive relationship of CD4^+^ T-cell increases with neutralization of Env entry in DC. A longitudinal analysis for repeated measures by generalized estimating equation method was used for the analysis

*CI* confidence interval

### Tat vaccination maintains CD4^+^ T cells and contains viral load rebound in patients non-compliant to therapy

**Fig. 9:**
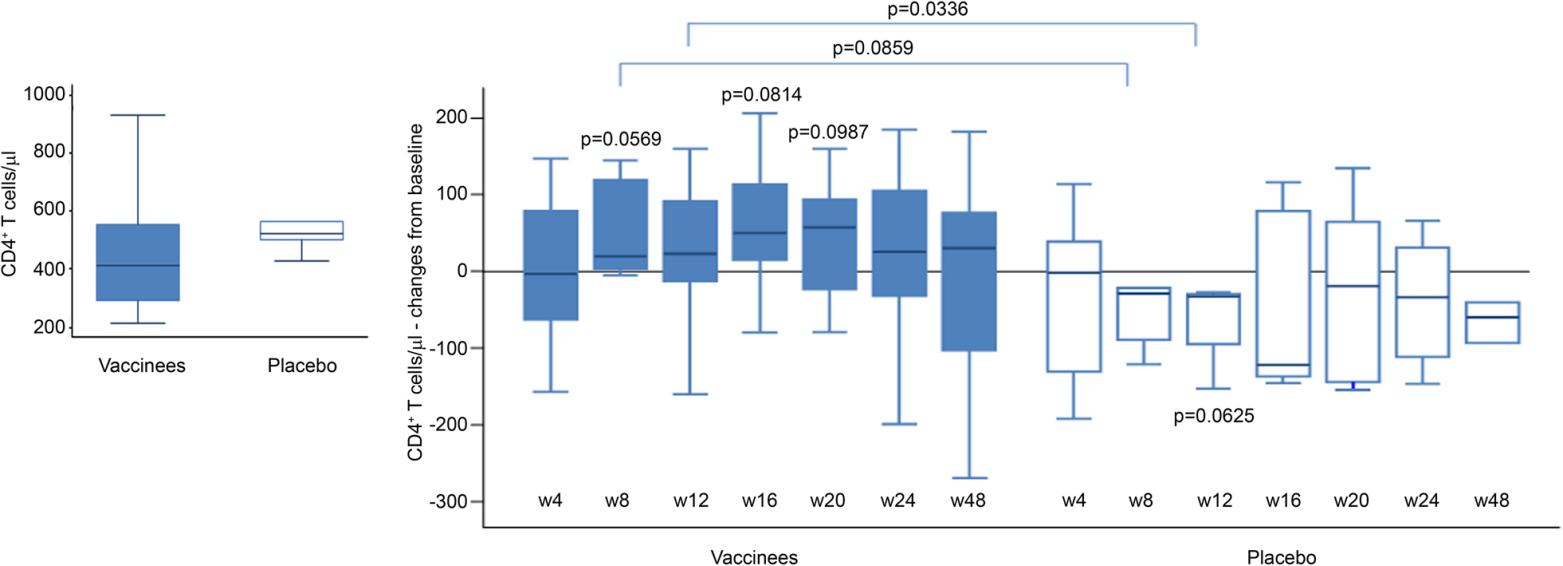
Changes from baseline of CD4^+^ T-cell number in vaccinees and placebos non compliant to therapy. Baseline values (*left panel*) and changes from baseline after immunization (*right panel*) of CD4^+^ T-cell counts in vaccinees (n = 18) and placebos (n = 5). Data are presented as *box plots*. Wilcoxon signed rank sum test for paired data and Wilcoxon–Mann–Whitney test were used for the analyses. p values assess the changes from baseline within and between treatment groups

Compliance was always verified at each study visit. However, despite counseling for adherence to therapy, medical records showed poor compliance (i.e. missing doses up to prolonged interruptions) in 24 volunteers, particularly between week 20 and 48 after the first immunization. Of them, 18 were vaccinees and 6 were placebos (1 anti-Tat Ab-positive and five anti-Tat Ab-negative). None of the vaccinees non-compliant to cART therapy experienced a decay of CD4^+^ T cells which, instead, increased above study entry levels (median increase of 50 cells/µL at week 16, p = 0.0814, 57 cells/µL at week 20, p = 0.0987 and 30 cells/µL at week 48, as compared to baseline). In contrast, the anti-Tat Ab-negative placebos had CD4^+^ T-cell decreases below study entry levels (median of −33 cells/µL at week 12, p = 0.0625 and −60 cells/µL at week 48 vs. baseline levels). Comparison between vaccinees and anti-Tat Ab negative placebos showed significant differences at week 8 and week 12 (p = 0.0859 and p = 0.0336, respectively) (Fig. 9).

**Fig. 10:**
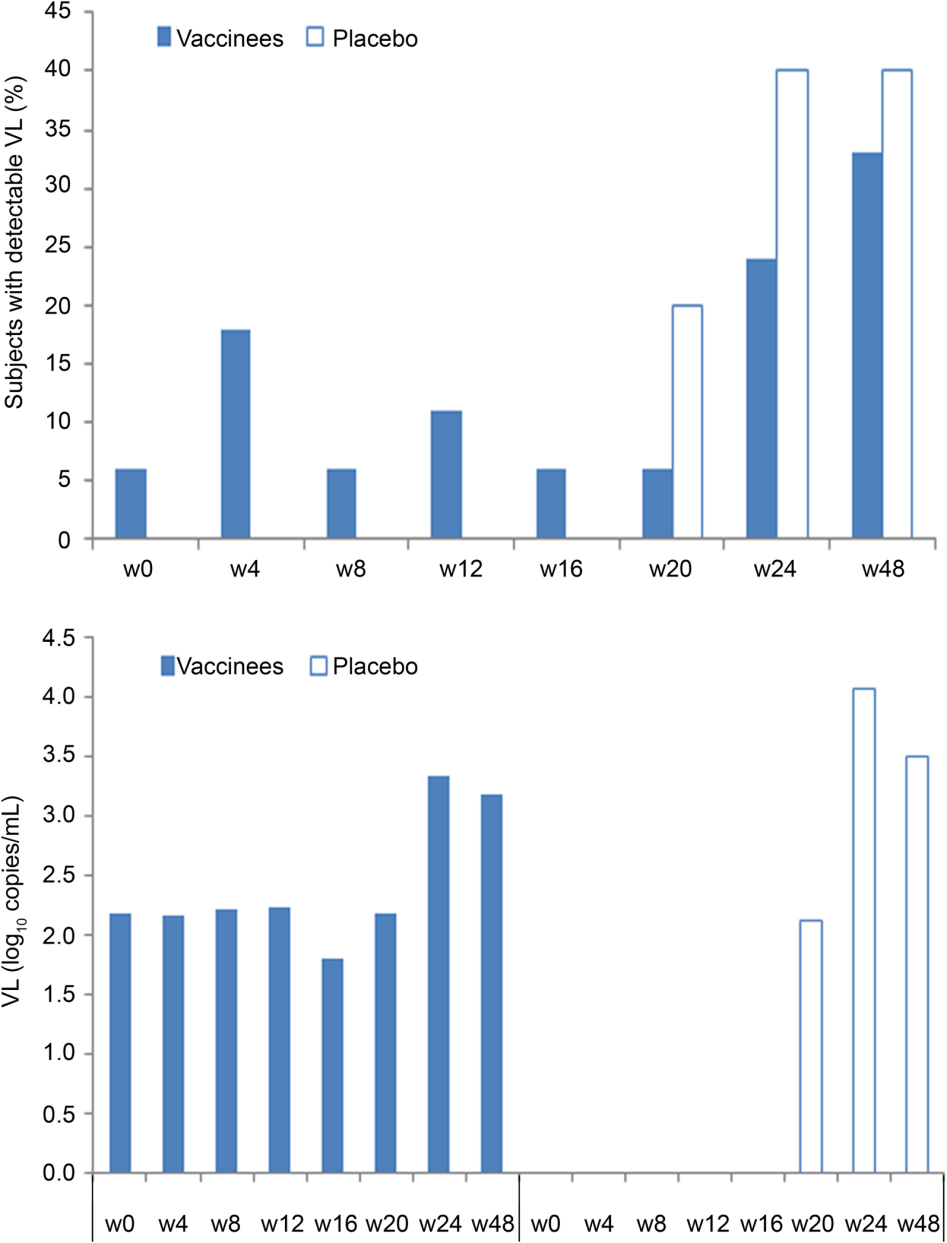
Plasma viremia up to week 48 in vaccinees and placebo non compliant to therapy. Percentage of vaccinees and anti-Tat Ab-negative placebos non-compliant to cART with detectable plasma viremia (*upper panel*), and plasma viremia values (log_10_ copies/mL) in patients with detectable viral load at each study visit (*lower panel*)

With regard to viral load, plasma viremia remained undetectable at week 48 in 12/18 (67 %) vaccinees, and in 3/5 (60 %) of anti-Tat Ab-negative placebos. In addition, in patients with detectable viral load at week 48, the geometric mean levels were lower in vaccinees (1090 copies/mL), as compared to anti-Tat Ab-negative placebos (3179 copies/mL) (Fig. 10).

## Discussion

The development of therapeutic vaccination strategies for treating people already infected with HIV-1 has been recently accelerated, with an increasing number of vaccine candidates being tested in clinical trials, either in drug-naïve patients or in association with cART. In drug-naïve patients, therapeutic vaccines are expected to contain infection (i.e. low to undetectable plasma viral load and CD4^+^ T cell preservation), preventing progression to disease as well as virus transmission, while in cART-treated patients therapeutic vaccination is expected to intensify the efficacy of cART, thus supporting a more effective immune restoration and virological control, particularly in poor immunological responders or cART non-compliant patients, preventing progression to AIDS-related as well as non AIDS-related diseases and virus transmission.

No therapeutic vaccines are currently market approved. However, the rapidly expanding HIV/AIDS therapeutic vaccine field portraits a variety of approaches, which differ sensibly in many aspects, the most relevant being the antigen chosen (unlike preventative vaccines, regulatory and accessory genes are frequently targeted; in some cases almost the entire HIV genome is targeted), and the delivery systems, which range from simple subcutaneous, intradermal, or intramuscular vaccine administration to reinfusion of autologous DCs loaded ex vivo with the selected antigen(s) [9, 79–89]. In most vaccine trials conducted in treated patients cART therapy was interrupted to assess the potency of the immunological control of infection provided by vaccination, while they were not aimed at evaluating the immunological recovery, with the exclusion of CD4^+^ T cell counts, which, however, did not appear to go beyond the restoration provided by cART alone [9, 79].

Our approach has focused on Tat, a key HIV virulence factor, which is released extracellularly in a biologically active form also under cART, and promotes virus reactivation, replication and spreading while inducing immune activation and disabling the host immune defense (reviewed in [25]). Thus, induction of effective anti-Tat Abs may represent a pathogenesis-driven therapeutic intervention to block disease progression as indicated by the effects of long-lasting, high titers anti-Tat Abs in natural infection [9, 79] or after vaccination with Tat, which induced CD4^+^ T cell recovery, immune restoration, as well as reduction of immunoactivation and of proviral DNA in Italian subjects (ISS T-002 trial) [53, 57].

The results of the present study indicate that B-clade Tat immunization is safe and well tolerated also in South African individuals infected with a different virus subtype. Further, vaccination induced anti-Tat Abs in almost all vaccinees. Abs were durable, at high titers and of multiple subclasses. Remarkably, B-clade Tat vaccination induced cross-clade (A, C, D) Tat-binding Abs, which were capable of neutralizing Tat-mediated entry in DC of oligomeric HIV Env from B and C clade, suggesting that the B-clade Tat protein used in our vaccine program may be used for a cross-clade HIV vaccine approach.

A natural humoral anti-Tat Ab response developed in a small number of placebos, a finding expected from previous studies conducted in Italy and South Africa [53, 54, 69, 70]. Indeed, as compared to the other HIV proteins which elicit Abs virtually in all infected patients, production of anti-Tat Abs is seen only in about 20 % of the HIV-infected subjects present in all cohorts we have investigated. Surprisingly, although Tat is released extracellularly, only a small percentage of individuals recognizes and mounts an Ab response against this protein [66–70]. One reason could be its molecular mimicry for extracellular matrix proteins such as fibronectin (FN) and vitronectin (VN) [90–92]. As for Tat, FN and VN possess a similar basic region and RGD sequence binding to the α5β1, αVβ3 and αVβ5 integrins [93, 94].

In vaccinees, neutralization correlated positively with anti-Tat IgM and IgG Ab titers, whereas Env entry was not neutralized by anti-Env Abs in the absence of anti-Tat Abs [57, 65]. Indeed, anti-Env Abs measured prior to immunization both in vaccinees and placebos had a positive correlation with increased levels of Tat-mediated Env entry in DC. This reproduces what has been seen earlier with sera from Italian vaccinees (ISS T-002 trial) or in monkey studies [57, 65]. In particular, by forming a complex with Env, Tat increases virus entry in DC and blocks neutralization by anti-Env Abs, which is restored and further increased only in the presence of anti-Tat Abs [65].

Tat vaccination was associated with significant increases of CD4^+^ T cells above baseline levels, whereas placebos showed a slower kinetics and lower, and not statistically significant, increases, as expected in individuals on cART for a mean of about 3 years. Increases of CD4^+^ T cells in vaccinees correlated significantly with neutralization. Of note, CD4^+^ T cells increased particularly in vaccinees with lower CD4^+^ T cell counts at baseline. This is of particular relevance since poor immunological response to therapy is frequent either in patients starting cART late, even if virologically suppressed [4–8], or in patients with persistent immune activation [95–99] or low compliant [100–103]. A poor CD4^+^ T cell recovery (<500 T cells/μL) is associated with disease progression, co-morbidities, hospitalization and death [104–107]. These patients are those that most require ART intensification.

While in the ISS T-002 trial conducted in Italy patients were highly compliant to therapy, compliance was lower in the ISS T-003 study, a finding particularly frequent in Southern Africa where scarce adherence to cART therapy represents a relevant clinical problem since it is associated with disease progression, virus drug resistance and transmission [108–111]. Of interest, none of the vaccinees non-compliant to cART therapy experienced a decay of CD4^+^ T cells, and in most of them plasma viremia remained undetectable at week 48 while, in those with detectable viremia, viral load levels were low. In contrast, the anti-Tat Ab-negative placebos had CD4^+^ T-cell decreases below entry levels, and in most of them plasma viremia rebounded to geometric mean levels higher than those recorded in non-compliant vaccinees. Although these results are only descriptive, since the groups are too small to draw any firm conclusion, they indicate the need of ad hoc studies to address whether cART intensification by Tat therapeutic immunization may mitigate the effects of low adherence to therapy. To this end, structured therapy interruption studies after cART intensification by the Tat vaccine are being planned.

The results of the ISS T-003 trial are highly consistent with those of the ISS T-002 [53, 57], although the two trials were conducted in individuals with different genetic background, infected with HIV from different subtypes (B vs. C clade), and on cART for different periods of time (i.e. mean of 6 years in the ISS T-002 trial vs. about 3 years in the ISS T-003 trial). Indeed, safety and immunogenicity results were remarkably similar, sometimes identical, as were the CD4^+^ T-cell increments, particularly in subjects with lower levels at baseline [53, 57], suggesting that poor immunological responders to therapy could greatly benefit from Tat immunotherapy.

The results from the Italian trial (ISS T-002) clearly indicate that proviral DNA reduction (as opposed to CD4^+^ T cell increase) is a late event, particularly under NNRTI-based drug regimens requiring 108 weeks for detecting a significant proviral reduction [57]. Indeed, no significant reductions of proviral DNA are seen at week 48 in both (ISS T-002 and ISS T-003) trials (data not shown). Furthermore, it appears that time on effective cART is also relevant, in that proviral DNA decay plateau after about 4–5 years of successful therapy [112, 113]. Thus, unlike the Italian trial in which vaccinees had been on therapy on average for 6 years, subjects enrolled in the South African study had been on cART for around 3 years. Altogether these data indicate that longer periods of time are required to see an effect on proviral DNA in NNRTI-treated South African subjects, which represent 97 % of the trial population.

Therefore, similarly to the ISS T-002 trial, a roll-over observational study (ISS T-003 EF-UP) has been initiated for the South African trial to ensure the extended follow-up of the volunteers, in order to evaluate the persistence of vaccine-induced immune responses as well as the immunological and virological effects of Tat immunization. In particular, proviral DNA will be monitored to verify whether Tat vaccination is capable of reducing it, as observed for the ISS T-002 trial after 3 years from vaccination [57].

## Conclusions

These data indicate that immunization with B clade Tat induced functionally effective cross-clade anti-Tat Abs and CD4^+^ T-cell increases and reinforce the notion that B clade Tat is a suitable candidate for therapeutic immunization against different HIV clades in different geographical areas, thus supporting the future conduct of phase III studies in South Africa.

## Additional files

10.1186/s12977-016-0261-1 B, C, A and D Tat clades sequences and GeneBank accession numbers. Based on data published in Hemelaar J et al. (AIDS 2011, 2:679-689), which refer to the time period 2004-2007, a frequency of 0.98, 27.28, 11.54, and 3.61 % for HIV-1 subtypes B, C, A and D, respectively, was calculated for the African continent.
10.1186/s12977-016-0261-1 ISS T-003 study protocol.

## Abbreviations

Ab: antibody
ABTS: 2,2′-azino-bis(3-ethylbenzothiazoline-6-sulphonic acid
AE: adverse event
cART: combined antiretroviral therapy
BSA: bovine serum albumin
CCR5: C–C chemokine receptor 5
DC: dendritic cell
DEAE: diethylaminoethyl
EDTA: ethylene diamine tetra-acetic acid
ELISA: enzyme-linked immunosorbent assays
Env: envelope
FITC: fluorescein isothiocyanate
GMP: good manufacturing practice
HCT: HIV counselling and testing campaign
HIV: human immunodeficiency virus
HLA-DR: human leukocyte antigen-D related
Ig: immunoglobulin
HSA: human serum albumin
min: minutes
MDDC: monocyte-derived dendritic cell
MedDRA: medical dictionary for regulatory activities
ND_50_: 50 % neutralization
NK: natural killer
NNRTI: non-nucleoside reverse-transcriptase inhibitors
NRTI: nucleoside reverse transcriptase inhibitors
OD: optical density
PBS: phosphate-buffered solution
PI: protease inhibitor
SAE: serious AEs
Tat: transactivator of transcription
WHO: World Health Organization
