# Supplementary figures and images for: HIV-Tat immunization induces cross-clade neutralizing antibodies and CD4+ T cell increases in antiretroviral-treated South African volunteers: a randomized phase II clinical trial
Source: Retrovirology. 2016 Jun 9;13:34. doi: 10.1186/s12977-016-0261-1 (PMC4899930; doi:10.1186/s12977-016-0261-1)

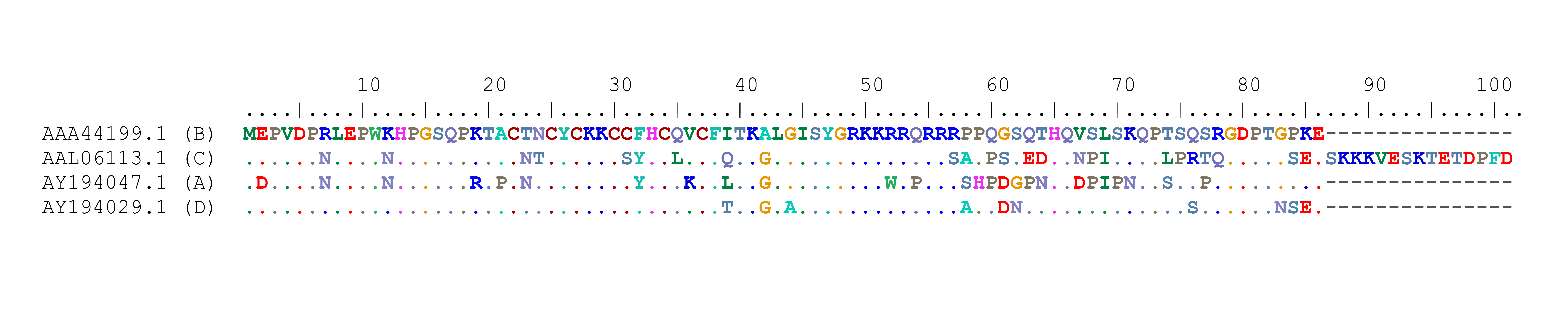

Supplement: Supplementary file 1 — 10.1186/s12977-016-0261-1 B, C, A and D Tat clades sequences and GeneBank accession numbers. Based on data published in Hemelaar J et al. (AIDS 2011, 2:679-689), which refer to the time period 2004-2007, a frequency of 0.98, 27.28, 11.54, and 3.61 % for HIV-1 subtypes B, C, A and D, respectively, was calculated for the African continent. [file 12977_2016_261_MOESM1_ESM.tif]
